# The Effects of Antipsychotics in Experimental Models of Krabbe Disease

**DOI:** 10.3390/biomedicines11051313

**Published:** 2023-04-28

**Authors:** Kapil Sharma, Kumlesh K. Dev

**Affiliations:** Drug Development Research Group, Department of Physiology, School of Medicine, Trinity College Dublin, D02 R590 Dublin, Ireland

**Keywords:** astrocytes, demyelination, haloperidol, antipsychotics, twitcher mice, psychosine, Krabbe disease, Schizophrenia

## Abstract

The role of altered myelin in the onset and development of schizophrenia and changes in myelin due to antipsychotics remains unclear. Antipsychotics are D_2_ receptor antagonists, yet D_2_ receptor agonists increase oligodendrocyte progenitor numbers and limit oligodendrocyte injury. Conflicting studies suggest these drugs promote the differentiation of neural progenitors to oligodendrocyte lineage, while others report antipsychotics inhibit the proliferation and differentiation of oligodendrocyte precursors. Here, we utilised in-vitro (human astrocytes), ex-vivo (organotypic slice cultures) and in-vivo (twitcher mouse model) experimental study designs of psychosine-induced demyelination, a toxin that accumulates in Krabbe disease (KD), to investigate direct effects of antipsychotics on glial cell dysfunction and demyelination. Typical and atypical antipsychotics, and selective D_2_ and 5HT_2A_ receptor antagonists, attenuated psychosine-induced cell viability, toxicity, and morphological aberrations in human astrocyte cultures. Haloperidol and clozapine reduced psychosine-induced demyelination in mouse organotypic cerebellar slices. These drugs also attenuated the effects of psychosine on astrocytes and microglia and restored non-phosphorylated neurofilament levels, indicating neuroprotective effects. In the demyelinating twitcher mouse model of KD, haloperidol improved mobility and significantly increased the survival of these animals. Overall, this study suggests that antipsychotics directly regulate glial cell dysfunction and exert a protective effect on myelin loss. This work also points toward the potential use of these pharmacological agents in KD.

## 1. Introduction

Schizophrenia is a chronic condition involving the complex interplay between genetic and environmental factors and is thought to be associated with aberrant neurodevelopment [1,2]. Schizophrenia is associated with abnormalities in grey and white matter, as well as loss of cerebral volume and anatomical pathology [3]. While neuronal dysfunction has been well studied in schizophrenia, the role of glial cells in the pathophysiology of this disease is still emerging, and, in particular, the role of altered oligodendrocyte biology is less clear [4]. Differing theories exist, although there is no consensus as to whether altered levels of myelin are linked with the onset of this illness, if altered myelination is a cause or consequence associated with the development of the condition, and/or how levels of myelin change during short and long-term use of antipsychotic treatment. There is an agreement, however, that patients with schizophrenia have decreased white matter volume and integrity, where post-mortem studies demonstrate white matter pathology linked to deficits in myelin, axons and mature oligodendrocytes [5,6]. In addition, altered expression and risk variants of several genes playing a role in oligodendrocyte maturation and myelination have also been associated with this illness, where variants in myelin-related genes increase schizophrenia susceptibility [7,8,9].

Antipsychotic treatment of patients with schizophrenia has also been suggested to directly regulate the expression of genes associated with oligodendrocyte maturation and myelination, thus influencing white matter integrity [5,6]. The effects of antipsychotics are, however, somewhat controversial, where studies suggest these drugs either promote proliferation and differentiation of neural progenitors to oligodendrocyte lineage or instead can inhibit differentiation of oligodendrocyte precursor cells [6,9]. Antipsychotics, such as clozapine, have been demonstrated to reduce neuroinflammation and demyelination in the experimental autoimmune encephalomyelitis (EAE) model of multiple sclerosis (MS) and enhance the rate of functional recovery in the cuprizone non-immune model of demyelination [10,11]. When considering the pharmacology of antipsychotics regulating levels of myelin, it is known that hyper-dopaminergia in the basal ganglia is linked with schizophrenia, where typical antipsychotics have dopamine D_2_ receptor subtype antagonism and atypical antipsychotics have additional serotonin (e.g., 5HT_2A_) receptor antagonism [12]. It has been reported that oligodendrocytes express dopamine D_2_ receptors, of which expression levels increase following combined oxygen and glucose deprivation [13]. Dopamine action on D_2_ receptors can affect myelin formation by regulating the development and function of oligodendrocytes [14,15]. In addition, dopamine receptors have been shown to play a role in the development of EAE and MS [16]. Paradoxically to typical antipsychotics being D_2_ receptor antagonists, studies show that D_2_ agonists increase the number of oligodendrocyte progenitor cells and protect oligodendrocytes against oxidative injury [13], which may explain the therapeutic effects of some antipsychotics that are partial D_2_ receptor agonists. Moreover, studies suggest the protective effect of D_2_ agonists can be diminished by D_2_ antagonists, suggesting that D_2_ receptor activation may play an important role in oligodendrocyte protection against injury, whereas typical antipsychotics may restrict oligodendrocyte recovery from injury. Contradictory, haloperidol and clozapine prevented apoptotic cell death in oligodendrocytes that were cultured under glucose-deprived conditions, highlighting their potential protective effects [17].

Krabbe disease (KD), also known as globoid cell leukodystrophy, is a rare autosomal recessive disease caused by mutations in the *galc* gene, which codes for the enzyme galactosylceramidase (GALC). Psychosine (galactosylsphingosine) is a toxic lipid that accumulates abnormally in those with KD. It has been shown to be toxic to oligodendrocytes and cause demyelination by various proposed mechanisms [18]. Given the uncertainty of the effects of antipsychotics on glial cells and myelin state, this study examined the direct effects of multiple typical and atypical antipsychotics on human astrocyte cell toxicity, tested the archetypical drugs haloperidol and clozapine on levels of myelin in mouse organotypic cerebellar slices, and lastly demonstrated effects of haloperidol in a demyelinating mouse model, specifically the twitcher mouse model of KD. The aims of this study were to investigate if antipsychotics prevented psychosine-induced toxicity in human astrocytes, psychosine-mediated demyelination in organotypic slices, and improve survival, twitching scores, immobility scores and subtle behavioural metrics on open field testing in a murine model of psychosine toxicity, namely the twitcher mouse model of KD.

## 2. Materials and Methods

### 2.1. Compounds and Antibodies

Compounds were reconstituted in dimethyl sulfoxide (DMSO, Sigma, Darmstadt, Germany; D8418) at stock concentrations of 5 mM and 20 mM depending on solubility, and were as follows: Clozapine (Cayman, Ann Arbor, MI, USA; 12059), Olanzapine (Cayman, Ann Arbor, MI, USA; 11937), Amisulpride (Cayman, Ann Arbor, MI, USA; 14619), Quetiapine (Acros organics, Geel, Belgium; 462400050), Risperidone (TCI, Tokyo, Japan; R0087), Aripiprazole (TargetMol, Wellesley Hills, MA, USA; T1566), Haloperidol (TCI, Tokyo, Japan; H0912), Chlorpromazine (TargetMol, Wellesley Hills, MA, USA; T1384), Sulpiride (TargetMol, Wellesley Hills, MA, USA; T1201) Eticlopride (Merck, Darmstadt, Germany; E101) and Volinanserin (Merck, Darmstadt, Germany; M3324). Psychosine (ChemCruz, Begonialaan, The Netherlands; sc-202781A) was prepared as 10 mM stock concertation in DMSO. Working concentrations from stock compounds were made using serum-free media prior to treatments. Primary antibodies (1:1000 dilution) were mouse anti-vimentin (Vimentin, Santa-Cruz, Heidelberg, Germany; Sc-373717; AB10917747), chicken anti-glial fibrillary acidic protein (GFAP, Abcam, Cambridge, UK; ab4674; AB304558), mouse anti-myelin oligodendrocyte glycoprotein (MOG, Millipore, Darmstadt, Germany; MAB5680; AB1587278), rabbit anti-myelin basic protein (MBP, Abcam, Cambridge, UK; ab40390; AB1141521), chicken anti-neurofilament H (NFH, Millipore, Darmstadt, Germany; ab5539; AB177520), rabbit anti-ionized calcium binding adaptor molecule 1 (Iba1, Wako, Neuss, Germany; 019-19741; AB839504) and mouse anti-neurofilament H non-phosphorylated (anti-SMI32, Millipore, Darmstadt, Germany; NE1023; AB2715852). Secondary antibodies (1:1000 dilution) were goat Alexa-549 anti-mouse (Jackson immune research, Cambridge, UK; 115-5060-068), goat Alexa-633 anti-chicken (Invitrogen, Waltham, MA, USA; A21103) and goat Alexa-488 anti-rabbit (Invitrogen, Waltham, MA, USA; A11008). Hoescht (4 µM) was used to stain cell nuclei (Thermo Scientific, Waltham, MA, USA; 62249).

### 2.2. Human Astrocytes Cell Culture Studies

Human astrocytes (ScienCell Research Laboratory, Carlsbad, CA, USA; Cat No. #1800, Lot No. 9063) were cultured exactly as we have described previously [19,20,21,22,23,24,25,26,27,28]. Confluent cells were cultured in serum-free media for four hours and then treated as specified in Figure legends. For MTT and LDH assays, human astrocytes were seeded in 96 well plates at a density of 0.01 × 10^6^ per well and cultured for 24 h until greater than 80% confluent. For MTT assays, after treatment, media was removed and replaced with 100 µL of fresh serum-free media supplemented with 10 µL of 12 mM MTT Formazan (Sigma, Darmstadt, Germany; m2003) and plates were incubated for 2.5 h at 37 °C. Subsequently, 75 µL of media was removed, and 50 µL of DMSO was added per well. Cells were incubated for ten minutes, and the absorbance was read at 540 nm. For LDH assays, cytotoxicity was measured on aliquots of the cellular supernatant using the CyQUANTTM LDH Cytotoxicity Assay Kit following manufacturer instructions. LDH activity was measured using 490-nm absorbance. The immunocytochemistry and fluorescent microscopy of human astrocytes was carried out as we have outlined previously [19,20,21,22,23,24,25,26,27,28]. Images of cells were acquired using scanning confocal microscopy (Leica, Ashbourne, Ireland; SP8) at 10× and 20× magnification. The number of astrocyte projections of 20–30 cells per treatment group was analysed using ImageJ software.

### 2.3. Mouse Organotypic Cerebellar Slice Culture Studies

Organotypic cerebellar slice cultures were prepared exactly as we have described previously [21,29,30,31]. At 12 days in vitro (DIV), slices were treated as per Figure legends, and slices were prepared for immunohistochemistry at 14 DIV. Immunohistochemistry and confocal fluorescent microscopy were also carried out, as we have outlined previously [19,20,21,22,24,26,28,30,32]. Cerebellar slices were washed with PBS and then fixed with 4% PFA for 10 min. Slices were then blocked and permeabilised with 10% BSA and 0.05% triton-x in PBS overnight. Slices were then incubated for 48 h in an appropriate primary antibody diluted in 2% BSA + 0.1% triton-x and then washed and incubated for 24 h in an appropriate secondary antibody. The slices were rinsed and placed on glass microscope slides with SlowFade^®^ (Invitrogen, Waltham, MA, USA; s36936), and the edges were closed with varnish. Slides were stored in the dark until imaged. Images of slices were acquired using scanning confocal microscopy (Leica, Ashbourne, Ireland; SP8) at 10× and 20× magnification. The mean fluorescence for each treatment group was analysed by examining regions of interest (ROI). Image analysis was carried out using ImageJ (https://imagej.nih.gov/ij/) and Imaris^®^ software.

### 2.4. Twitcher Model In Vivo Studies

All animal work was carried out in compliance with EU legislation approved by Trinity College Dublin ethics committee and in accordance with guidelines from the Health Products Regulatory Authority (HPRA) under project authorisation number AE19136/P123. A colony of heterozygous twitcher mice obtained from Jackson Laboratory, Cambridge, UK (B6.CE-Galctwi/J Stock no:000845) were maintained for breeding under pathogen-free conditions. Ear punch samples were genotyped by TransnetYX (www.transnetyx.com) using real-time PCR. Haloperidol was given at a dose of 1 mg/kg/day in drinking water. Mice were kept in grouped cages, had constant access to food and water and were under a 12-h light/dark timetable. Humane endpoints, weight measurements, behavioural observations, twitching, immobility and locomotor scores were all as per established protocols previously published by our group [18]. Blinding was not conducted/possible for drug treatment or genotype as twitcher mice phenotype became observable over time. Data were pseudo-anonymised by using animal numbers, and analysis was carried out blinded to genotype and treatment. For open field maze (OFM) testing, animals were habituated on post-natal day (PND) 21 and 22 for 5 min. On PND 25, 28 and 30, animals were tested before the emergence of severe twitching or immobility. Animals were placed in the centre of the OFM apparatus, a 44 cm × 44 cm cage with highly darkened walls, for 5 min. Video recordings were analysed using ANYmaze tracking software (Stoelting), examining; distance (m), mean speed (m/s), max speed (m/s), time mobile (%), centre entries and corner time (%).

### 2.5. Statistical Analysis

Statistical analysis was performed using GraphPad Prism 9 (GraphPad Software, Inc., San Diego, CA, USA), and the values shown are means +/− standard error of the mean. The sample size was calculated for one-way ANOVA by referencing previously published astrocyte psychosine toxicity experiments by our group [21,23,24,31]. A range of estimated mean change and standard deviation was set as 20–25% and 5–8%, respectively. A power of 0.8 and a stricter than conventional alpha of 0.1 was utilised to ensure experiments were not underpowered. Each experimental replicate or “*n*” for cultured human astrocytes were cells at different passages performed in triplicate. Normality was assessed by generating normal QQ plots for data and assessing for any obvious skewness. Clear outliers where identified were removed. Formal normality tests were carried out using D’Agostino-Pearson or Anderson-Darling tests for experiments with sufficient ‘n’ numbers. In experiments with smaller but still suitably powered sample sizes, normality was assessed using Shapiro-Wilk or Kolmogorov-Smirnov tests. Parametric one-way or two-way ANOVA tests or non-parametric Kruskal-Wallis tests followed by Tukey’s or Dunn’s multiple comparisons tests, respectively, were carried out for experiments comparing means from selected groups. Survival analysis was carried out using the Kaplan-Meier curve with a log-rank Mantel-Cox test. Further information on statistical methods is given in the results section and in Figure legends. In all cases, the significance levels (alpha) were fixed at *p* < 0.05 *, *p* < 0.01 **, *p* < 0.001 *** and *p* < 0.0001 ****.

## 3. Results

### 3.1. Antipsychotics Prevent Psychosine-Induced Toxicity in Human Astrocytes

**Figure 1:**
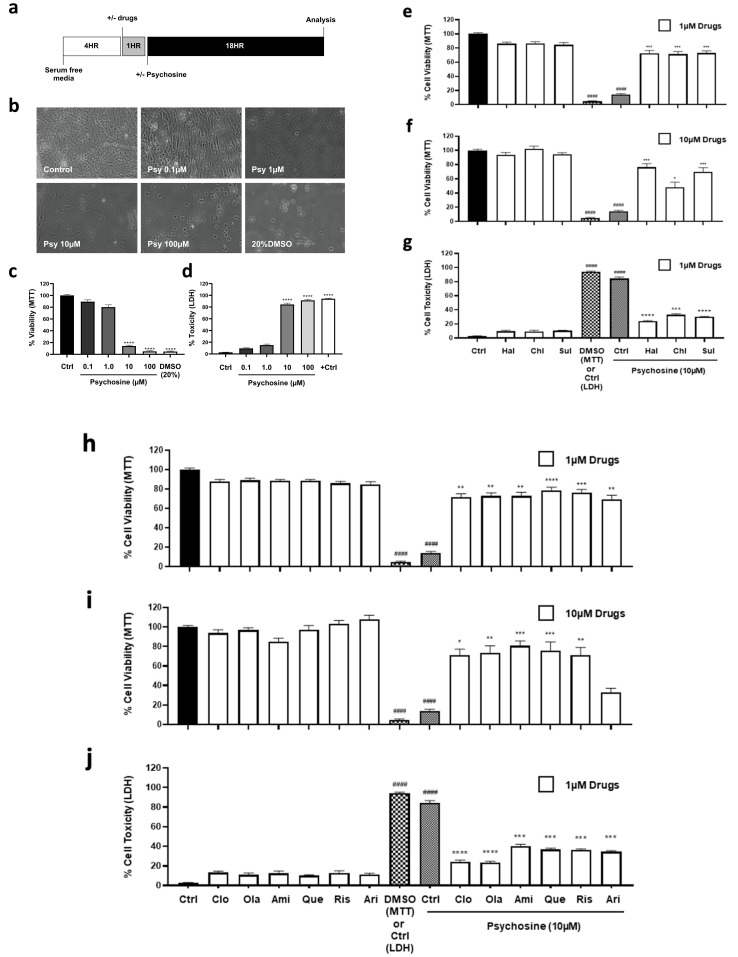

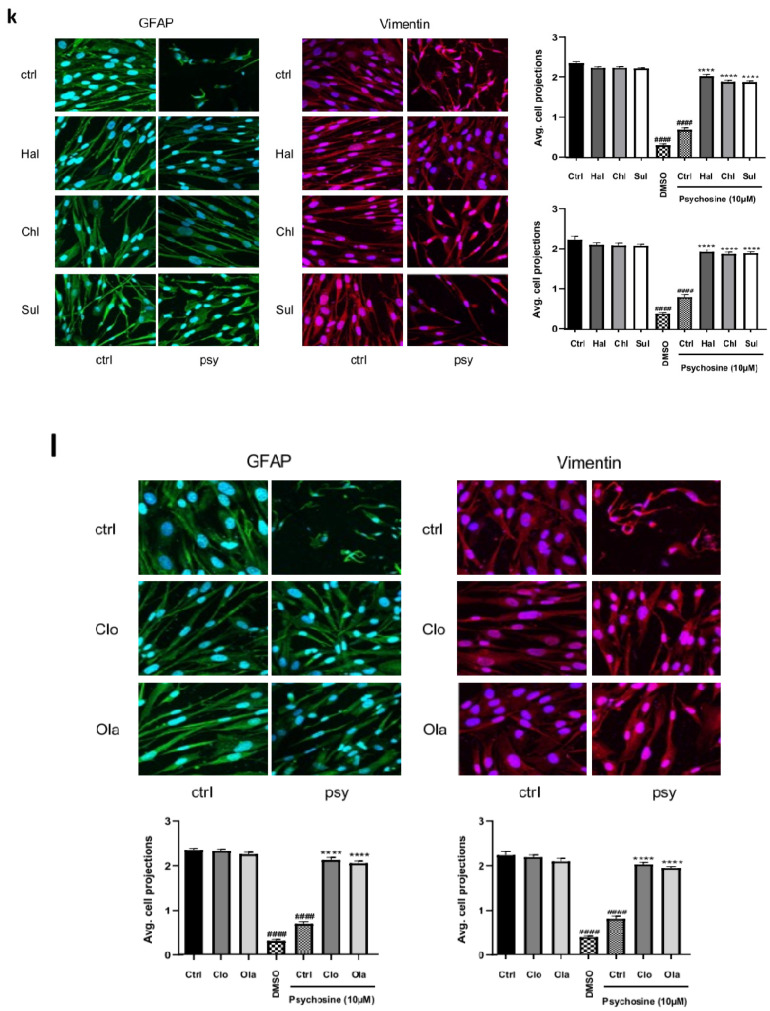
Antipsychotics prevent psychosine toxicity of human astrocytes. (**a**) The experimental schematic diagram for treatment timelines prior to analysis. (**b**) Light microscopy images of astrocytes. (**c**) MTT assays of psychosine-induced cell toxicity in human astrocytes. Positive control of 20% DMSO. Kruskal-Wallis, Dunn’s multiple comparison tests (*n* = 5–10). (**d**) LDH assays of psychosine-induced cell toxicity in human astrocytes. One-way ANOVA, Tukey’s multiple comparison tests (*n* = 5). MTT assays showed that typical (**e**) 1 µM or (**f**) 10 µM and atypical (**h**) 1 µM or (**i**) 10 µM antipsychotics did not alter cell viability compared to control, while DMSO (20%) and psychosine (10 µM) significantly reduced cell viability compared to control (*p* < 0.0001 ^####^). All antipsychotics (1 µM or 10 µM) (except aripiprazole (Ari) at 10 µM) significantly prevented psychosine (10 µM) induced reductions in cell viability (*p* < 0.05 *, *p* < 0.01 **, *p* < 0.001 *** and *p* < 0.0001 ****). Kruskal-Wallis, Dunn’s multiple comparison tests (*n* = 5–10). LDH assays showed that (**g**) typical and (**j**) atypical antipsychotics at 1 µM were not toxic to cells compared to control, whereas DMSO (20%) and psychosine (10 µM) significantly increased cell toxicity compared to control. All antipsychotics (1 µM) prevented psychosine (10 µM) toxicity. One-way ANOVA, Tukey’s multiple comparison test (*n* = 5). (**k**) Typical antipsychotics and (**l**) atypical antipsychotics (1 µM) had no effect alone on astrocyte morphology compared to control, while DMSO (20%) and psychosine (10 µM) reduced the number of astrocyte extensions. (**k**) Typical antipsychotics and (**l**) atypical antipsychotics (1 µM) attenuated morphological changes induced by 10 µM psychosine. Representative confocal cell fluorescent images labelled for GFAP (green), Vimentin (red) and Hoescht (blue). The number of astrocyte projections of 20–30 cells per treatment group was analysed. Images were analysed using ImageJ software. One-way ANOVA, Tukey’s multiple comparison tests (*n* = 6). Typical antipsychotics included: Haloperidol (Hal), chlorpromazine (Chl) and sulpiride (Sul). Atypical antipsychotics included: clozapine (Clo), olanzapine (Ola), amisulpride (Ami), quetiapine (Que), risperidone (Ris) and aripiprazole (Ari).

**Figure 2:**
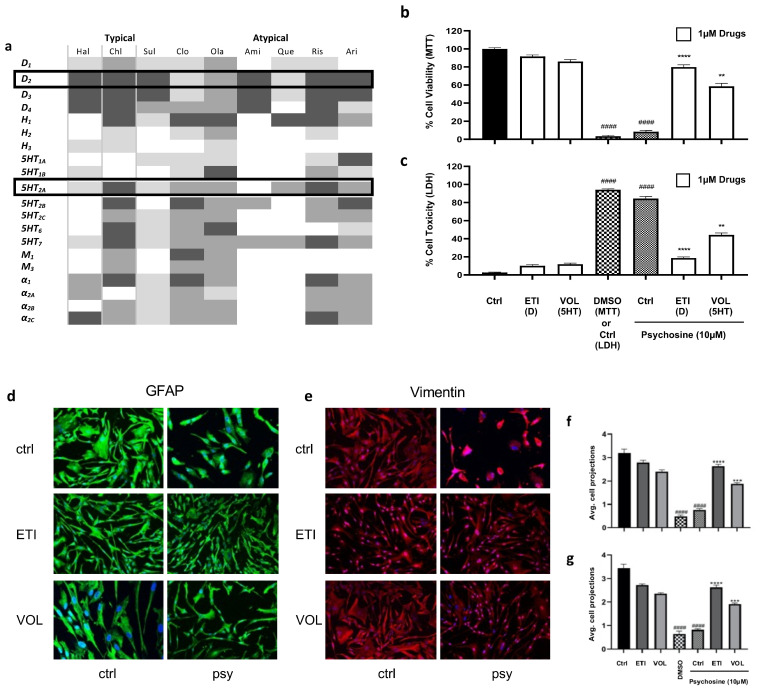
Selective D_2_ and 5HT_2A_ antagonists prevent psychosine-induced toxicity. (**a**) Pharmacology Heat Map. Receptor binding profiles of antipsychotics are shown. Inhibitory constant (Ki): 100 nM < Ki < 10,000 nM (weak, light grey), 10 nM < Ki < 100 (moderate, mid grey), 1 > Ki < 10 (strong, dark grey). All drugs are antagonists or inverse agonists at D2 and 5HT2A receptors apart from aripiprazole which is a partial agonist at these receptors. Haloperidol (Hal), chlorpromazine (Chl), sulpiride (Sul), clozapine (Clo), olanzapine (Ola), amisulpride (Ami), quetiapine (Que), risperidone (Ris) and aripiprazole (Ari) (**b**) MTT assays showed that 1 µM Eticlopride (ETI) (selective D_2_ antagonist) and 1 µM Volinanserin (VOL) (selective 5HT_2A_ antagonist) did not alter cell viability compared to control, while DMSO (20%) and psychosine (10 µM) significantly reduced cell viability compared to control. Both 1 µM Eticlopride (ETI) and 1 µM Volinanserin (VOL) prevented significant reductions in cell viability induced by 10 µM psychosine Kruskal-Wallis, Dunn’s multiple comparison tests (*n* = 5). (**c**) LDH assays showed that 1 µM Eticlopride (ETI) and 1 µM Volinanserin (VOL) did not induce cell toxicity compared to the control. In contrast, DMSO (20%) and psychosine (10 µM) significantly increased cell toxicity compared to the control (####). Both drugs prevented significant cell toxicity induced by 10 µM psychosine. One-way ANOVA, Tukey’s multiple comparison tests (*n* = 5). Representative confocal cell fluorescent images labelled for (**d**) GFAP (green) and (**e**) Vimentin (red), with Hoescht (blue) staining. Eticlopride (ETI) and Volinanserin (VOL) at 1 µM had no effect alone on astrocyte morphology compared to control, while DMSO (20%) and psychosine (10 µM) reduced the number of astrocyte extensions. Both selective antagonists attenuated morphological changes induced by 10 µM psychosine. The number of (**f**) GFAP (green) and (**g**) Vimentin (red) positive astrocyte projections of 20–30 cells per treatment group were analysed. Images were analysed using ImageJ software. One-way ANOVA, Tukey’s multiple comparison tests (*n* = 6). *p* < 0.01 **, *p* < 0.001 *** and *p* < 0.0001****.

Human astrocytes were serum starved for 4 h and then treated with or without antipsychotics for 1 h before the addition of psychosine for 18 h, after which cells were analysed (Figure 1a). Light microscopy (Figure 1b), MTT (Figure 1c) and LDH assays (Figure 1d) showed that psychosine caused a concentration-dependent loss of astrocyte numbers, as did the 20% DMSO positive control. MTT (Figure 1e,f) and LDH (Figure 1g) assays of typical antipsychotics showed 1 µM or 10 µM haloperidol, chlorpromazine, or sulpiride prevented significantly psychosine (10 µM) induced reductions in cell viability. Similarly, using MTT (Figure 1h,i) and LDH (Figure 1j) assays, a range of atypical antipsychotics at 1 µM or 10 µM significantly prevented toxicity induced by 10 µM psychosine, including clozapine, olanzapine, amisulpride, quetiapine, risperidone and aripiprazole. Immunocytochemistry using expression of GFAP and Vimentin allowed for assessment astrocyte morphology and analysis of the average number of cellular projections beyond 50µm. Psychosine (10 µM) (and 20% DMSO) decreased number of cell projections, which was attenuated by the exemplary typical antipsychotics (1 µM) haloperidol, sulpiride, and chlorpromazine (Figure 1k) and the selected representative atypical antipsychotics clozapine and olanzapine (Figure 1l). Antipsychotics display antagonist and/or inverse agonist activity at D_2_ and 5HT_2A_ receptors, except aripiprazole which is a partial agonist (Figure 2a). Therefore, highly selective antagonists for D_2_ (Eticlopride) and 5HT_2A_ (Volinanserin) were used to determine their effects on psychosine-induced toxicity. MTT (Figure 2b) and LDH (Figure 2c) assays showed Eticlopride (1 µM) or Volinanserin (1 µM) significantly attenuated cell viability induced by 10 µM psychosine, with Eticlopride showing greater improvement relative to Volinanserin. Eticlopride or Volinanserin also significantly prevented the psychosine-induced reduction in GFAP (Figure 2d,f) and vimentin-positive projections (Figure 2e,g).

### 3.2. Haloperidol and Clozapine Attenuate Psychosine Induced Demyelination in Slice Cultures

**Figure 3:**
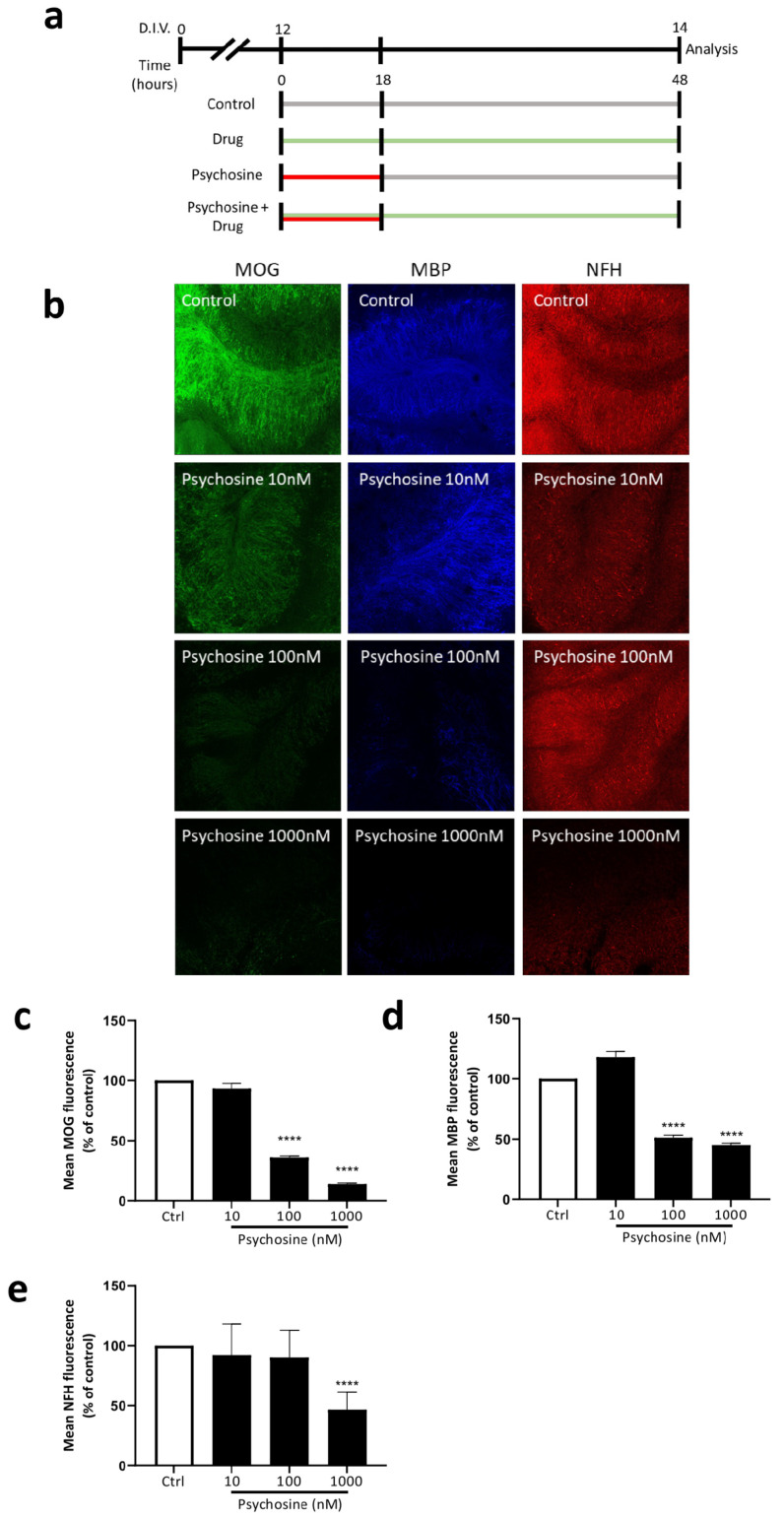

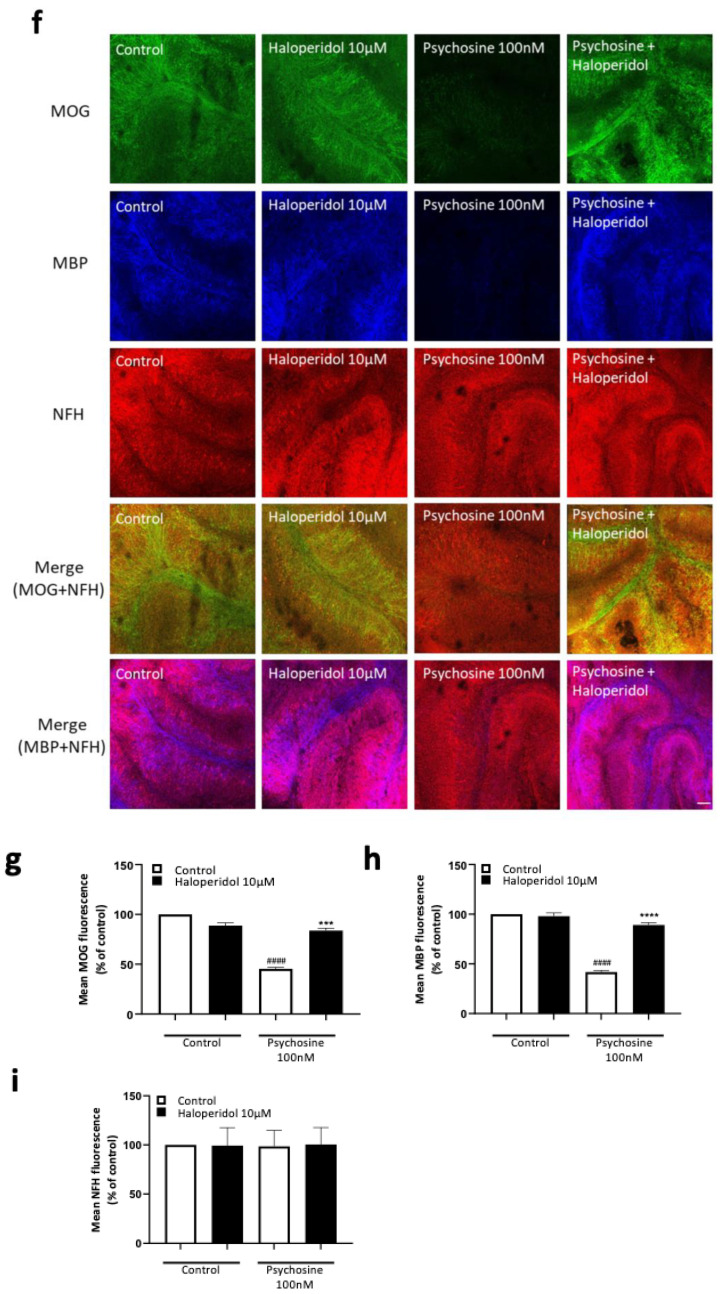

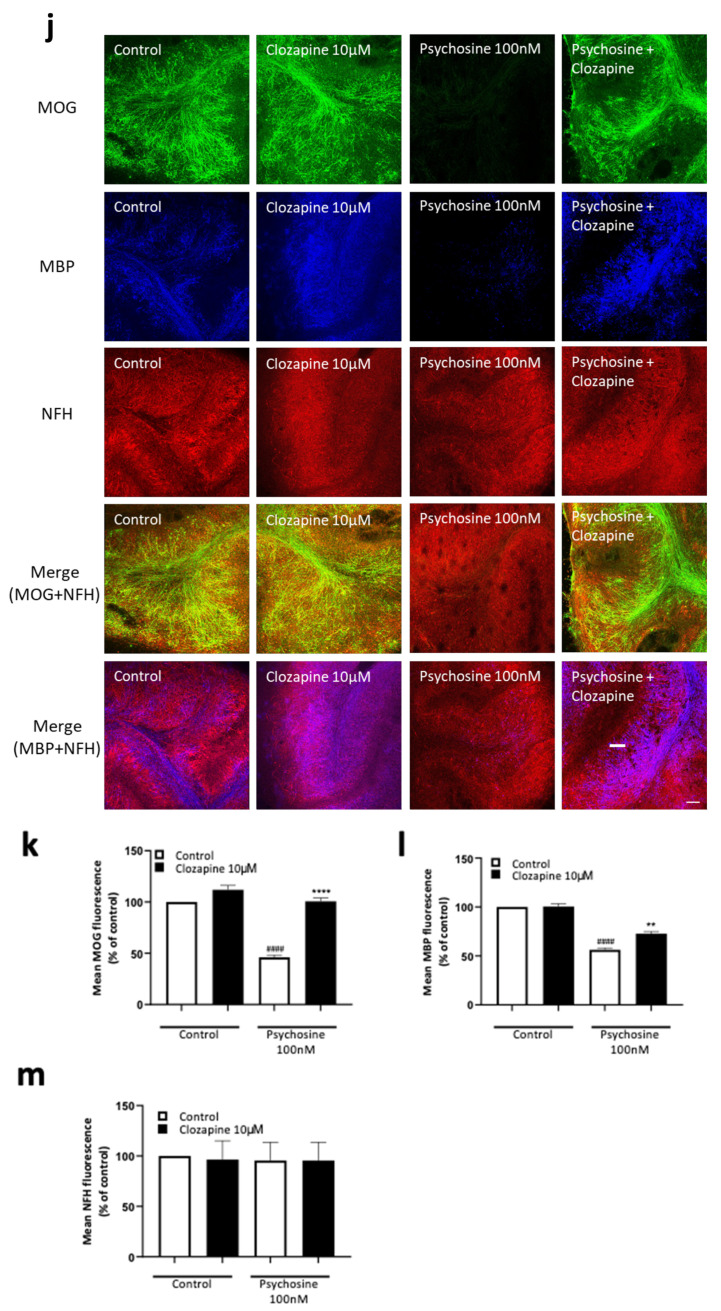
Antipsychotics attenuate psychosine-induced demyelination in organotypic cerebellar slices. (**a**) Experimental design schematic, cerebellar slices from 10-day-old C57BL/6J mice were cultured 12 days in-vitro (DIV) before treatment with or without psychosine and/or antipsychotics. Immunohistochemical analysis was performed at 14 DIV. (**b**) Representative confocal images showing the effect of Psychosine. A decrease in (**c**) MOG and (**d**) MBP fluorescence was seen at 100 nM and 1000 nM concentrations of psychosine. (**e**) NFH fluorescence was reduced at 1000 nM but not 100 nM psychosine. (**f**) Representative confocal images showing the effects of Haloperidol. Psychosine 100 nM decreased (**g**) MOG and (**h**) MBP, but not (**i**) NFH, fluorescence compared to control that was attenuated by Haloperidol 10 µM. (**j**) Representative confocal images showing the effects of Clozapine. Psychosine 100 nM decreased (**k**) MOG and (**l**) MBP, but not (**m**) NFH, fluorescence compared to the control that was attenuated by Clozapine 10 µM. Confocal images at 10× magnification, scale bar 100µm. Data are shown as mean +/− SEM, Kruskal-Wallis test, Dunn’s multiple comparisons test, ^####^ *p* < 0.0001, **** *p* < 0.0001, *** *p* < 0.001, ** *p* < 0.01 (*n* = 5–10).

**Figure 4:**
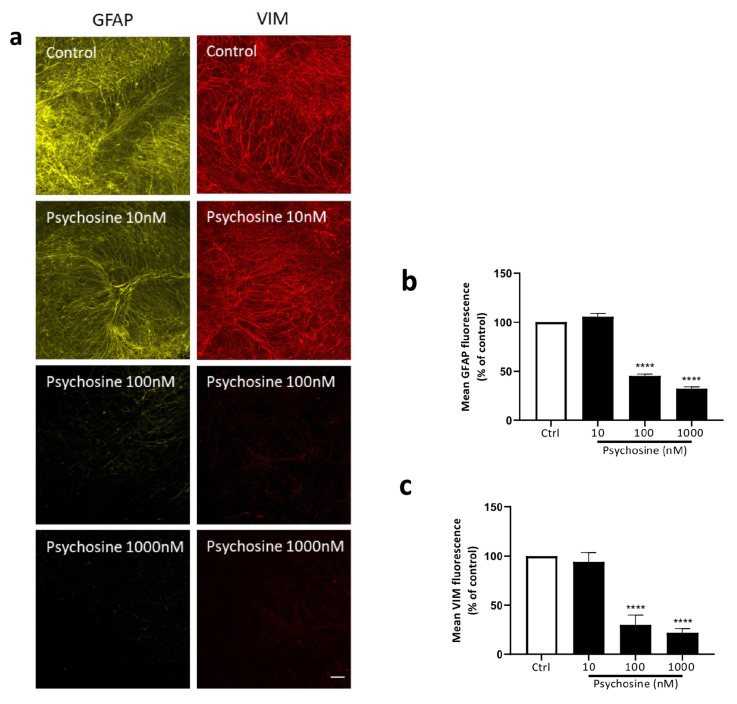

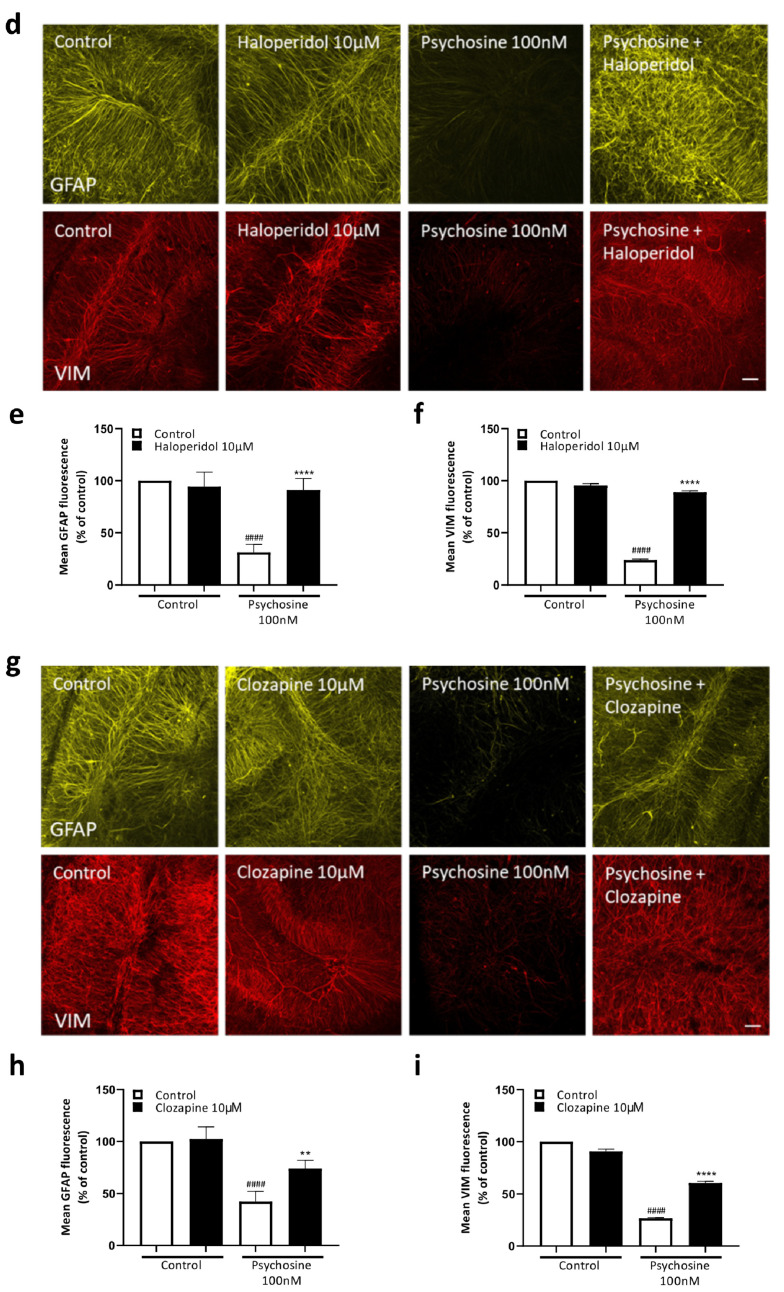

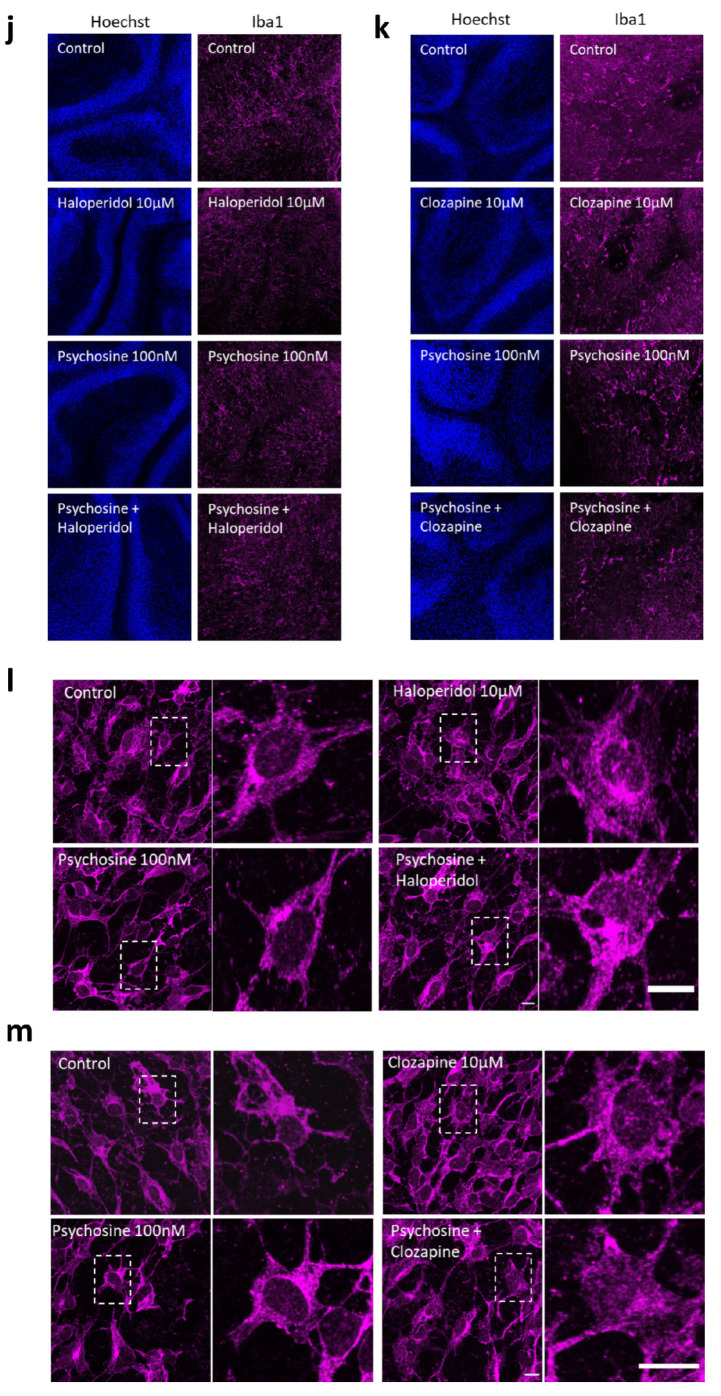
Psychosine induces changes in astrocytes, but not microglia, markers attenuated by Haloperidol and Clozapine. Treatment of cerebellar slices with psychosine decreases (**a**,**b**) GFAP and (**a**,**c**) Vimentin fluorescence at 100 nM and 1000 nM psychosine. Haloperidol 10 µM attenuated psychosine-induced decrease in (**d**,**e**) GFAP and (**d**,**f**) Vimentin fluorescence at 100 nM psychosine. Clozapine 10 µM attenuated the psychosine-induced decrease in (**g**,**h**) GFAP and (**g**,**i**) Vimentin fluorescence at 100 nM psychosine. Psychosine in the presence or absence of (**j**,**l**) Haloperidol or (**k**,**m**) Clozapine did not alter (**j**,**k**) Iba1 fluorescence or (**l**,**m**) microglia morphology in white matter tracts or whole cerebellar slices areas. Confocal images at 10× and 20× magnification, scale bar 100 µm and 10 µm, respectively. Data are shown as mean +/− SEM, Kruskal-Wallis test, Dunn’s multiple comparisons tests, ^####^ *p* < 0.0001, **** *p* < 0.0001 and ** *p* < 0.01 (*n* = 5).

**Figure 5:**
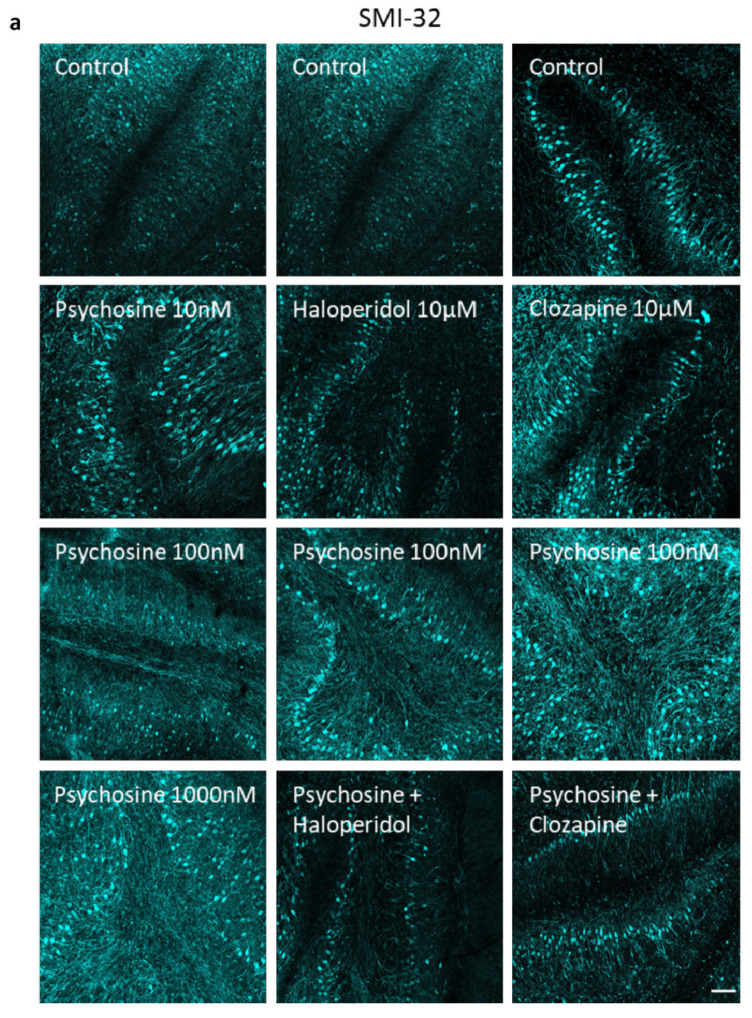

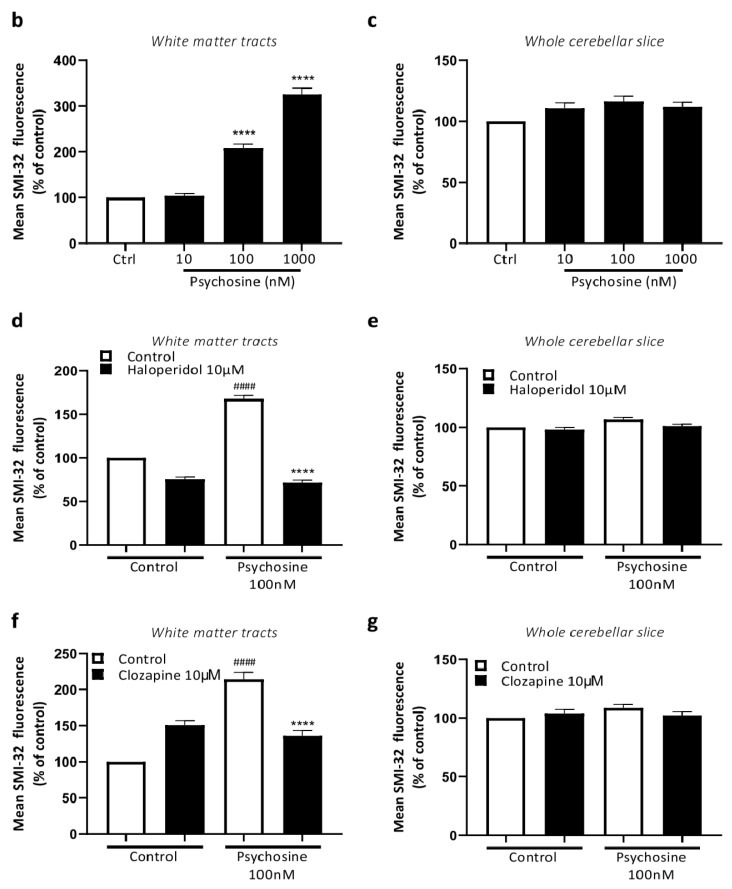
Psychosine-induced increase in SMI-32 fluorescence is attenuated by Haloperidol and Clozapine. (**a**) Representative confocal images of SMI32. Increase in SMI-32 fluorescence at 100 nM and 1000 nM psychosine in (**b**) white matter tracts of cerebellar slices, without change in (**c**) whole cerebellar slice areas. Increase in SMI-32 fluorescence at 100 nM psychosine in white matter tracts of cerebellar slices were attenuated by (**d**) Haloperidol 10 µM and (**f**) Clozapine 10 µM, with no change in whole cerebellar slice areas by (**e**) Haloperidol 10 µM and (**g**) Clozapine 10 µM. Confocal images at 10× magnification, scale bar 100 µm. Data are shown as mean +/− SEM, Kruskal-Wallis test, Dunn’s multiple comparisons tests, **** *p* < 0.0001, ^####^ *p* < 0.0001 (*n* = 5).

Organotypic cerebellar slices were prepared from 10-day-old (P10) C57BL/6J mice, cultured for 12 DIV and treated with or without psychosine in the presence or absence of haloperidol or clozapine, with analysis conducted at 14 DIV (Figure 3a). Psychosine caused a concentration-dependent decrease, in both myelin markers MOG (Figure 3b,c) and MBP (Figure 3b,d), with a loss of NFH only at a high 1 µM concentration (Figure 3b,e). The psychosine (100nM) induced decrease in mean MOG and MBP fluorescence was attenuated by haloperidol 10 µM (Figure 3f–i) and Clozapine 10 µM (Figure 3j–m). In agreement with cellular studies (Figure 1), psychosine also showed a concentration-dependent decrease in the mean fluorescence of GFAP (Figure 4a,b) and vimentin (Figure 4a,c). The decrease in GFAP and vimentin fluorescence induced by 100 nM psychosine was attenuated by haloperidol 10 µM (Figure 4d–f) and clozapine 10 µM (Figure 4g–i), corroborating cellular studies (Figure 1). The psychosine-induced demyelination occurred without a change in microglia activation (Figure 4j,k) and morphology (Figure 4l,m), with no effect of haloperidol (Figure 4j,l) or clozapine (Figure 4k,m). Psychosine caused axonal damage in organotypic cerebellar slices as demonstrated by a concentration-dependent increase in mean SMI-32 fluorescence, specifically within white matter tracts (Figure 5a,b), with no global change in levels across the whole cerebellar slice (Figure 5c). The psychosine 100nM induced increase in mean SMI-32 fluorescence was attenuated by haloperidol 10 µM (Figure 5a,d) and clozapine 10 µM (Figure 5a,f), again specifically in the white matter tracts with no change across the whole cerebellar slice (Figure 5e,g).

### 3.3. Haloperidol Improves Survival in Twitcher Mice

**Figure 6:**
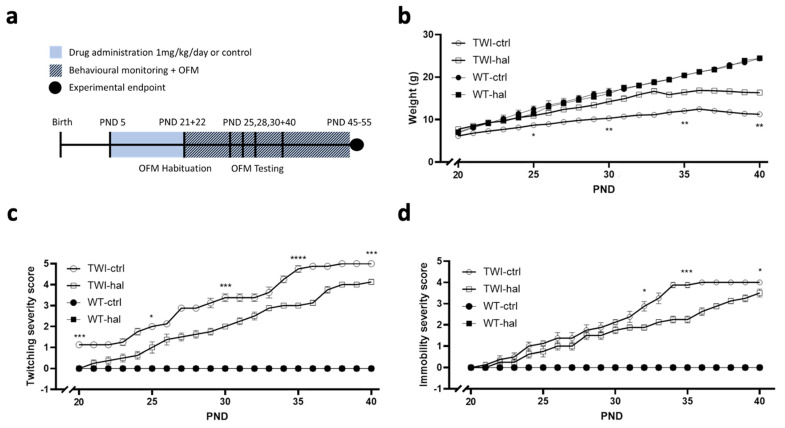

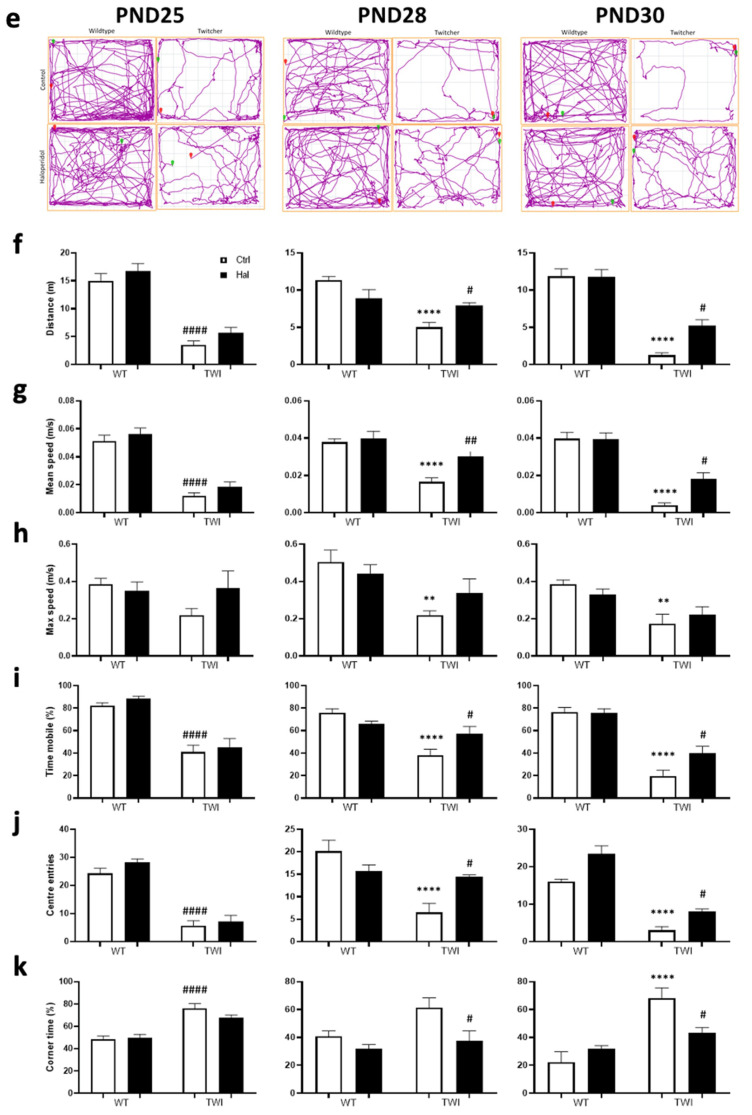

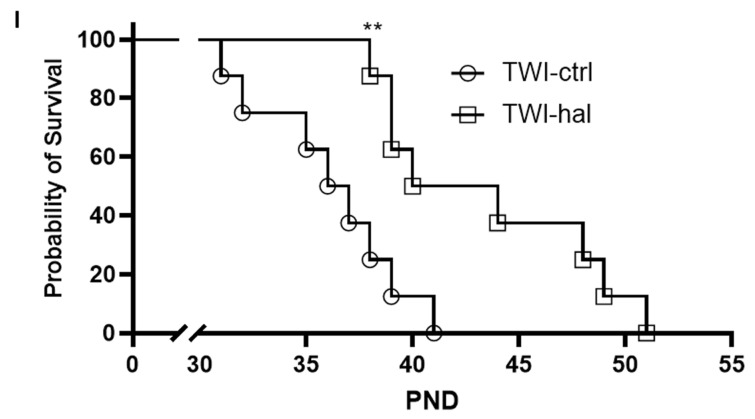
Haloperidol improved survival in twitcher mice. (**a**) Experimental design schematic. Homozygous mice and their wild-type litter mates were treated with haloperidol at a dose of 1 mg/kg/day in drinking water or just water control via a suckling pipette. (**b**) Weight for different treatment groups of wildtype and twitcher mice. Twitcher mice treated with haloperidol had significantly higher body weight at different time points compared to twitcher mice not treated with the drug. (**c**) Twitching severity and (**d**) immobility severity scores for different treatment groups of wildtype and twitcher mice. Twitcher mice treated with haloperidol had significantly improved twitching and immobility at different time points compared to twitcher mice not treated with the drug. Two-way ANOVA followed by Tukey’s multiple comparisons analysis demonstrated significant differences in (**b**) weight, (**c**) twitching score and (**d**) immobility score between haloperidol-treated and untreated twitcher mice. (* *p* < 0.05, ** *p* < 0.01, *** *p* < 0.001, **** *p* < 0.0001, *n* = 8 per group). Graphs show data as mean ± SEM. (**e**) Representative image of open field maze test carried out at PND 25, 28 and 30. Bar graphs for (**f**) distance (m), (**g**) mean speed (m/s), (**h**) max speed (m/s), (**i**) time mobile (%), (**j**) centre entries and (**k**) corner time (%). (^#^ *p* < 0.05, ^##^ *p* < 0.01, *^####^ p* < 0.0001). One-way ANOVA followed by Tukey’s multiple comparisons analysis (*n* = 8 per group). Graphs show data as mean ± SEM. (**l**) Survival analysis by Kaplan-Meier curve comparing haloperidol-treated twitcher mice versus untreated twitcher mice. A log-rank Mantel-Cox test showed that haloperidol-treated twitcher mice had a significantly increased lifespan compared to untreated twitcher mice (** *p* < 0.01, *n* = 8 per group). For behavioural metrics for each treatment group for PND 25, PND 28 and PND 30, please also see Table 1.

The typical antipsychotic haloperidol was given at a dose of 1 mg/kg/day to twitcher mice starting from PND5 onwards (Figure 6a). Haloperidol-treated twitcher mice had significantly increased body weight compared to untreated twitcher mice (Figure 6b). Twitching severity scores improved with the administration of haloperidol, showing a significantly slower progression of this behaviour over the course of the experiment (Figure 6c). Additionally, mobility scores deteriorated significantly less rapidly in haloperidol-treated twitcher mice compared to untreated twitcher mice during the experimental period (Figure 6d). As expected, no significant differences in twitching scores, mobility scores and body weight were observed in wild-type control of haloperidol treated animals. An open field maze test was conducted on PND 25,28,30 when twitching and immobility scores were expected to be mild to moderate. A computer video tracking system (ANYmaze—Stoelting) was utilised to record traces (Figure 6e), allowing analysis of the following metrics: distance (m) (Figure 6f); mean speed (m/s) (Figure 6g); max speed (m/s) (Figure 6h); time mobile (%) (Figure 6i); centre entries (Figure 6j) and corner time (%) (Figure 6k). Significant genotype differences were noted between wildtype and twitcher mice in all metrics by PND30 age (*p* values denoted as *). At PND 25, haloperidol showed no significant effect in twitcher mice on any measurement tested. At PND 28 and PND 30, haloperidol treatment induced subtle but significant (*p* values denoted as #) improvements in distance (m) (Figure 6f); mean speed (m/s) (Figure 6g); time mobile (%)(Figure 6i); centre entries (Figure 6j) and corner time (%)(Figure 6k), but not max speed (m/s) (Figure 6h) in twitcher mice. Lastly, Kaplan-Meier survival curves were generated to assess the survival rates of twitcher mice. Haloperidol increased the lifespan of twitcher mice when compared with vehicle treatment (Figure 6l) (Log-rank Mantel-Cox test ** *p* = 0.0041, Median survival: TWI_Hal_ 42 days; TWI_Ctrl_ 36.5 days, *n* = 8 per group).

## 4. Discussion

### 4.1. The Regulation of Glia Cells by Antipsychotics

Structural and functional abnormalities in astrocytes, oligodendrocytes, and microglia, as well as glial progenitor cells, have been proposed in Schizophrenia [4,33]. Antipsychotics reduce grey and white matter and regulate all glial cell types, although some contradictory studies are noted, including: (i) Oligodendrocytes express D_2_ receptors [13], with dopamine regulating myelin formation and the development and function of oligodendrocytes [14,15]. While agonists of D_2_ receptors increase oligodendrocyte progenitor cell numbers and protect oligodendrocytes against oxidative injury [13], the D_2_ antagonists haloperidol and clozapine are also shown to prevent apoptotic cell death in oligodendrocytes cultured under glucose deprived conditions [17]. Haloperidol and clozapine are protective in the inflammatory demyelinating EAE model of multiple sclerosis and the non-immune cuprizone model of demyelination [10,11]. (ii) Astrocyte subtypes are also regulated by antipsychotics [34], which enhance astroglial glutamatergic transmission [35], regulate cytokine expression [36], activate Cx43 channel activity [37] and alter a number of other signalling pathways in these cells. Antipsychotics improve disturbed metabolism in schizophrenia via dopamine receptors [38], enhance the release of D-serine from astrocytes [39] and reduce glutamate uptake in these cells [40]. Contrastingly, some studies highlight the concern that chronic antipsychotic use may contribute to progressive grey matter loss, perhaps by preferentially targeting astrocytes [41]. (iii) Microglia activation and release of inflammatory mediators is regulated by typical and atypical drugs [42,43,44]. Animal studies are controversial, with some suggesting antipsychotics increase Iba1 expression [45], while others show reduced microglial activation [46]. Lastly, drugs aiming to reduce microglial activation (minocycline) [47] and cyclooxygenase-2 (COX-2) inhibitors [48] have been proposed as effective treatment strategies for schizophrenia.

### 4.2. Psychosine Toxicity as a Model of Glial Cell Dysfunction

In this study, we used psychosine-induced in vitro and in vivo models to investigate the effects of antipsychotics on glial cell dysfunction and demyelination. Psychosine is a toxin that aggregates in the brains of those afflicted with the neurodegenerative disorder globoid cell leukodystrophy, Krabbe disease (KD) [49,50]. KD is a rare condition associated with progressive demyelination that mainly presents during infancy, but juvenile and adult presentations are possible. The mechanisms by which psychosine induces demyelination remain unclear; however, they may include (i) apoptotic processes and caspase-dependent pathway activation [51,52,53,54,55], (ii) accumulation in lipid rafts associated with regional cholesterol increases and inhibition of PKC activity [49,56,57,58] (iii) generation of LPC and arachidonic acid with the regulation of secreted phospholipase A2 (sPLA2) [52], and/or (iv) phosphorylation of neurofilament proteins reducing radial growth of axons, axonal defects and neuronal cell death [59,60]. Psychosine also negatively affects astrocyte viability, possibly via apoptotic processes and is also proposed to be pro-inflammatory [33,35,36,52]. It is possible that astrocytic reactivity may contribute a central role to the pathogenesis of KD [61], supported by data showing that oligodendrocytes from twitcher mice can myelinate axons when transplanted into the shiverer mouse model of demyelination [62].

### 4.3. Limitations and Future Directions

We acknowledge that our *in-vitro* human astrocyte studies relied on MTT assays of cell viability, and there are confounding factors that can influence such assays [63]. To address this and to improve the robustness of the data, we carried out additional LDH assays of cell toxicity, as well as morphological studies. We note, however, that future studies could examine the potential mechanisms by which antipsychotics might exert the protective effects seen, e.g., the use of pertussis toxin, selective protein kinase B (AKT), and extracellular signal-regulated kinase (ERK) inhibitors could be utilised to further analyse the potential downstream signalling effects of antagonism of these receptors on psychosine induced toxicity in human astrocytes. Our *ex-vivo* slice culture experiments used the cerebellum, as it is rich in white matter and myelin-producing oligodendrocytes, and this area has been shown to be prone to psychosine-induced toxicity in previous studies [21,23,24,29]. In future studies, however, the use of other brain regions, as well as investigation of non-neuronal cells, such as inflammatory adaptive and innate immune cells, as well as cell-cell interactions, would be interesting. We also note that our twitcher mice in-vivo experiment used haloperidol, and future studies could use other antipsychotics to add weight to the suggestion here that antipsychotics may promote survival in twitcher mice and be a potential novel therapy for KD. Further studies, and in particular, neuroimaging studies using antipsychotics or drugs with similar pharmacology in animal models of KD, may highlight their possible use in future human trials. Lastly, we note that the twitcher mouse model used in this study is severe, modelling infantile KD, where antipsychotics may initially limit or slow demyelination, but where fatality ensues ultimately likely due to multi-organ toxicity caused by high concentrations of psychosine. The investigation of novel therapies in late-onset disease may be of interest, as well as combinatorial approaches that include limiting the production of psychosine, enhancement of its removal, as well as protecting against demyelination.

### 4.4. Conclusions

Here we show that psychosine (galactosylsphingosine) induces cell toxicity and reduces cell viability in human astrocytes, in agreement with previous studies [21,23,24,29]. We demonstrate that typical and atypical antipsychotics attenuate this psychosine-induced cell toxicity, cell viability and morphological changes in human astrocytes. Pharmacological analysis shows antipsychotics commonly act as antagonists or inverse agonists of dopamine and/or serotonin receptors. Our data show that both the selective D_2_ antagonist (Eticlopride) and 5HT_2A_ antagonist (Volinanserin) reduce psychosine-induced toxicity and morphological changes in human astrocytes, with inhibition at D_2_ receptors showing stronger efficacy. We also show that haloperidol and clozapine attenuate psychosine-induced demyelination in cultured organotypic cerebellar slices and reduce psychosine-induced loss of GFAP and vimentin expression. Regarding microglia, in agreement with previous studies [21,23,24,29], we saw little effect of psychosine on Iba1 expression, although we note Iba1 is not a specific marker of altered microglia reactivity. Investigating neuronal and axonal damage, we observed psychosine increases the expression of SMI-32 in the white matter axonal tracts of the arbour vitae, in agreement with our previous studies [21,23,24,29]. Haloperidol and clozapine treatment prevent the axonal expression of SMI-32, thus demonstrating a level of protection against psychosine-induced axonal damage in white matter tracts of cultured cerebellar slices. Lastly, to translate these findings into an in vivo setting, we show that haloperidol ameliorates weight loss, improves twitching, immobility and locomotor metrics in twitcher mice, and that haloperidol improves the survival of twitcher mice. To our knowledge, this study is the first to examine the direct effects of such a broad selection of commonly prescribed typical and atypical antipsychotics in both in vitro and in vivo models of psychosine-induced toxicity and demyelination.

The main points presented here are the following: (i) antipsychotics with dopamine and serotonin antagonism directly regulate glial cell dysfunction and exert a protective effect on myelin loss in cell cultures and organotypic cerebellar slices, and (ii) haloperidol, an antipsychotic with mainly dopamine antagonism improved survival and had a positive effect on behaviour in twitcher mice, an established demyelinating murine model of KD. Taken altogether, our findings indicate that antipsychotics are myelin protective and suggest the potential therapeutic benefit of agents with similar antipsychotic pharmacology in Krabbe disease.

## Figures and Tables

**Table 1:** Behavioural metrics for each treatment group for PND 25, PND 28 and PND 30.

	WT^Ctrl^	WT^Hal^	TWI^Ctrl^	TWI^Hal^	WT^Ctr^ vs. WT^Hal^	WT^Ctr^ vs. TWI^Ctrl^	WT^Ctr^ vs. TWI^Hal^	WT^Hal^ vs. TWI^Ctrl^	WT^Hal^ vs. TWI^Hal^	TWI^Ctrl^ vs. TWI^Hal^
Metric	Mean ± SEM	Mean ± SEM	Mean ± SEM	Mean ± SEM						
No. of animals	8	8	8	8						
**Behavioural metrics for each treatment group for PND 25**										
Distance (m)	15.00 ± 1.3	16.81 ± 1.29	3.54 ± 0.70	5.67 ± 0.97	ns	**** *p* < 0.0001	**** *p* < 0.0001	**** *p* < 0.0001	**** *p* < 0.0001	ns
Mean speed (cm/s)	5.11 ± 0.43	5.61 ± 0.49	1.19 ± 0.24	1.89 ± 0.32	ns	**** *p* < 0.0001	**** *p* < 0.0001	**** *p* < 0.0001	**** *p* < 0.0001	ns
Max speed (cm/s)	38.53 ± 3.06	35.04 ± 4.67	21.88 ± 3.53	36.65 ± 8.90	ns	ns	ns	ns	ns	ns
Time mobile (%)	82.15 ± 2.67	88.33 ± 2.35	40.95 ± 6.07	45.08 ± 7.87	ns	**** *p* < 0.0001	**** *p* = 0.0002	**** *p* < 0.0001	**** *p* < 0.0001	ns
Centre entries	24.25 ± 1.88	28.25 ± 1.19	5.63 ± 1.82	7.25 ± 2.05	ns	**** *p* < 0.0001	**** *p* < 0.0001	**** *p* < 0.0001	**** *p* < 0.0001	ns
Corner time (%)	48.48 ± 2.86	49.95 ± 2.83	76.03 ± 4.28	67.60 ± 2.57	ns	**** *p* < 0.0001	*** *p* = 0.0013	**** *p* < 0.0001	* *p* = 0.003	ns
**Behavioural metrics for each treatment group for PND 28**										
Distance (m)	11.31 ± 0.53	8.92 ± 1.14	5.01 ± 0.65	7.93 ± 0.38	ns	**** *p* < 0.0001	* *p* = 0.0146	** *p* = 0.004	ns	* *p* = 0.0412
Mean speed (cm/s)	3.78 ± 0.18	3.99 ± 0.38	1.66 ± 0.22	3.01 ± 0.30	ns	**** *p* < 0.0001	ns	**** *p* < 0.0001	ns	*** *p* = 0.0099
Max speed (cm/s)	50.41 ± 6.43	44.26 ± 4.74	21.86 ± 2.38	33.86 ± 7.51	ns	** *p* = 0.0063	ns	* *p* = 0.0407	ns	ns
Time mobile (%)	75.68 ± 3.88	65.89 ± 2.53	38.16 ± 5.27	57.14 ± 6.45	ns	**** *p* < 0.0001	*p* = 0.0477	** *p* = 0.0017	ns	* *p* = 0.0412
Centre entries	20.13 ± 2.43	15.63 ± 1.46	6.50 ± 2.02	14.38 ± 0.46	ns	**** *p* < 0.0001	ns	** *p* = 0.0052	ns	* *p* = 0.0181
Corner time (%)	40.81 ± 3.99	32.06 ± 2.93	61.56 ± 6.87	37.53 ± 7.24	ns	ns	ns	** *p* = 0.0043	ns	* *p* = 0.0241
**Behavioural metrics for each treatment group for PND 30**										
Distance (m)	11.87 ± 1.00	11.77 ± 0.10	1.26 ± 0.32	5.22 ± 0.80	ns	**** *p* < 0.0001	**** *p* < 0.0001	**** *p* < 0.0001	**** *p* < 0.0001	* *p* = 0.0107
Mean speed (cm/s)	3.98 ± 0.33	3.95 ± 0.33	0.41 ± 0.11	1.81 ± 0.33	ns	**** *p* < 0.0001	**** *p* < 0.0001	**** *p* < 0.0001	*** *p* = 0.0001	* *p* = 0.0114
Max speed (cm/s)	38.45 ± 2.32	33.09 ± 2.83	17.18 ± 5.25	22.28 ± 4.09	ns	** *p* = 0.0025	* *p* = 0.0264	* *p* = 0.0296	ns	ns
Time mobile (%)	76.66 ± 3.98	75.72 ± 3.81	19.76 ± 5.11	39.77 ± 6.25	ns	**** *p* < 0.0001	**** *p* < 0.0001	**** *p* < 0.0001	**** *p* < 0.0001	* *p* = 0.0346
Centre entries	16.00 ± 0.66	23.50 ± 2.10	3.00 ± 1.02	8.00 ± 0.73	ns	**** *p* < 0.0001	*** *p* = 0.0007	**** *p* < 0.0001	**** *p* < 0.0001	* *p* = 0.0439
Corner time (%)	22.24 ± 7.52	32.22 ± 1.92	68.09 ± 7.49	43.58 ± 3.56	ns	**** *p* < 0.0001	ns	*** *p* = 0.0007	ns	* *p* = 0.0241

Behavioural Metrics at PND25—No significant difference was observed between haloperidol-treated twitcher mice and untreated twitcher mice in the distance, mean speed, max speed, time mobile, centre entries or corner time. Additionally, no significant difference was observed in haloperidol-treated and untreated wild-type mice. Significant differences were observed between twitcher mice and wild-type littermates. Behavioural Metrics at PND28—Significant differences were observed between haloperidol-treated twitcher mice and untreated twitcher mice in distance, mean speed, time mobile, centre entries or corner time. No significant difference was observed for max speed between haloperidol-treated and untreated twitcher mice. No significant difference was observed in haloperidol-treated and untreated wild-type mice. Significant differences were observed between twitcher mice and wild-type littermates. Behavioural Metrics at PND30—Significant differences were observed between haloperidol-treated twitcher mice and untreated twitcher mice in distance, mean speed, time mobile, centre entries or corner time. No significant difference was observed for max speed between haloperidol-treated and untreated twitcher mice. No significant difference was observed in haloperidol-treated and untreated wild-type mice. Significant differences were observed between twitcher mice and wild-type littermates. One-way ANOVA followed by Tukey’s multiple comparisons analysis (* *p* < 0.05, ** *p* < 0.01, *** *p* < 0.001, **** *p* < 0.0001, *n* = 8 per group).

## Data Availability

The data that support the findings of this study are available from the corresponding author upon reasonable request.
